# Neutrophil Gelatinase-Associated Lipocalin 2 Accelerates Hypoxia-Induced Endothelial Cell Injury via eNOS/NRF2 Signalling 

**DOI:** 10.22074/cellj.2021.7167

**Published:** 2021-08-29

**Authors:** Yang Gu, Wei Sun, Zhuo Xu, Jing Wang, Xiao Hu, Zhou-Zhou Lu, Zhang Xi-Wen

**Affiliations:** 1. Department of Cardiology, The Affiliated Huaian No. 1 Peopleâ€™s Hospital of Nanjing Medical University, Huai'an, Jiangsu, China; 2. Department of Cardiology, The First Affiliated Hospital of Nanjing Medical University, Nanjing, Jiangsu, China

**Keywords:** Endothelial Cells, Endothelial Nitric Oxide Synthase, Neutrophil Gelatinase-Associated Lipocalin 2, Nuclear Factor Erythroid-2-Related Factor 2

## Abstract

**Objective:**

Neutrophil gelatinase-associated lipocalin (NGAL), a lipocalin, is implicated in many cardiovascular diseases (CVD). The effect of NGAL on endothelial cells (ECs), particularly on ECs injured because of hypoxia, is unclear. In this study, we aim to explore the effect of NGAL in an EC injury in response to hypoxia.

**Materials and Methods:**

In this experimental study, we isolated and cultured mouse heart ECs (MHECs). The EC injury model was established by exposure of the ECs to hypoxia for 24 hours. The ECs were treated with NGAL (30, 60, 120, 250 and 500 ng/ml). Cell inflammation and oxidative stress were detected by corresponding assays. Apoptotic cells were stained by the terminal deoxynucleotidyl transferase dUTP nick end labelling (TUNEL) assay.

**Results:**

NGAL increased the inflammatory response at the baseline level and further augmented the hypoxia-induced inflammation response. Reactive oxygen species (ROS) levels increased upon NGAL treatment, which caused antioxidase/oxidase imbalance. NGAL also exaggerated hypoxia-induced oxidative stress. The cell apoptosis rate also increased in both the NGAL-treated normoxic and hypoxic conditions. NGAL also reduced endothelial nitric oxide synthase (eNOS)-nitric oxide (NO) signalling, thus decreasing the expression and nuclear translocation of nuclear factor erythroid-2-related factor 2 (NRF2), which was confirmed by overexpression of NRF2.

**Conclusion:**

NGAL exaggerates EC injury in both normoxic and hypoxic conditions by inhibiting the eNOS-NRF2 pathway.

## Introduction

Heart tissue is composed of many types of cells that include mainly cardiomyocytes, fibroblasts, endothelial cells (ECs) and inflammatory cells (1). ECs play an important role in maintaining the normal blood supply of the heart (1, 2). During hypoxia in the heart, ECs undergo inflammation, oxidative stress and increased apoptosis. These factors cause a reduction in capillaries, decreased blood supply to the heart, and lead to cardiomyocyte apoptosis, interstitial fibrosis and eventual induction of heart failure (HF) (3, 4). Moreover, endothelium-derived small molecules and peptides, such as nitric oxide (NO), prostacyclin, angiotensin II (Ang-II) and endothelin-1 (ET-1) are other factors that influence cardiomyocytes, fibroblasts and inflammatory cells (5, 6). Hence, preventing EC injury during hypoxia and maintaining the normal function of ECs is of great importance.

NO is a small molecule peptide that can cause smooth muscle cells to relax (7). NO plays different roles at different concentrations. Low concentrations of NO exert positive inotropic effects on the heart and high concentrations of NO exert inotropic negative effects (6). NO is synthesized by three different NO synthases (NOS) - endothelial NOS (eNOS), inducible NOS (iNOS) and neuronal NOS (nNOS) (8). Production of NO by eNOS has protective effects in many models of cardiovascular diseases (CVD) such as cardiac hypertrophy (9), myocardial infarction (MI) (10) and cardiac ischemia/reperfusion injury (11). The results of studies show that eNOS-NO activates nuclear factor erythroid-2-related factor 2 (NRF2) and performs anti-oxidative stress, anti-apoptosis and cardiac-protective effects (12). Thus, targeting the eNOS-NO pathway in ECs may be a promising therapeutic method for EC injury during hypoxia or other cardiac diseases.

Neutrophil gelatinase-associated lipocalin (NGAL), an acute phase protein released by neutrophils, has been described as a biomarker of many CVD. NGAL is elevated in patients with type 2 diabetes who present with carotid artery stenosis (13). NGAL is involved in renal injury associated with the progression of HF (14) and cardiovascular events in patients with stable coronary artery disease (15). NGAL is a biomarker for the detection of unstable carotid plaques in asymptomatic patients (16). In animal experiments, NGAL has been reported to mediate post-MI cardiac damage by activating the NF-kappa B pathway (17). NGAL promotes airway remodelling in chronic obstructive pulmonary disease (18) and exaggerates cardiac hypertrophy and HF (19). These reports indicate the potential role of NGAL in EC injury. Therefore, this study aims to explore the functional role of NGAL in a hypoxia-induced EC injury and its underlying mechanisms.

## Materials and Methods

### Mouse heart endothelial cell (MHEC) isolation and culture

In this experimental study, MHECs isolation was referred to a previous study protocol (20). The mouse hearts were removed and cut into pieces and washed with Hanksâ€™ balanced salt solution buffer. Heat tissue was then digested with Collagenase A. Dulbeccoâ€™s modified Eagleâ€™s medium (DMEM, C11995, Thermo Fisher Scientific, USA) with 10% foetal bovine serum (FBS, 10099141, Thermo Fisher Scientific, USA) was used to stop the digestion process and the cells were subsequently filtered through a nylon mesh (70 mm pores). Then, the cells were bound to CD31 beads. The MHECs were cultured in dishes precoated with 2% gelatin (Sigma, Oakville, ON, Canada). The MHECs were cultured in FBS-free DMEM for 12 hours, then cultured in various concentrations of NGAL (30, 60, 120, 250 or 500 ng/ml) for 24 hours, followed by exposure to hypoxic conditions for 24 hours. The hypoxia model was described previously (21). Cell in the hypoxia group were placed in a BioSpherix C-Chamber (5% oxygen) for 24 hours. Cells in the control group were cultured in 5% CO_2_ and 95% air at 37ËšC. In order to test the effect of NGAL on cell viability, we divided the cells into six groups: control, 30 ng/ml NGAL, 60 ng/ml NGAL, 120 ng/ml NGAL, 250 ng/ml NGAL and 500 ng/ml NGAL. In order to detect the effect of NGAL on EC hypoxia, the cells were divided into four groups: vehicle-normoxia, NGAL-normoxia (500 ng/ml), vehicle-hypoxia and NGAL-hypoxia (500 ng/ml). To overexpress NRF2, adenovirus (Ad)-NRF2 (Vigene Biotech, Shangdong, China) was transfected 6 hours after treatment with NGAL. The cells were divided into three groups: Ad-NC, NGAL (500 ng/ml) and NGAL+Ad-NRF2. Cell viability was detected by the 3-(4,5-dimethylthiazol-2- yl)-2,5-diphenyltetrazolium bromide (MTT) assay (Biotech, Shanghai, China).

All experiments were approved by the Institutional Animal Care and Use Committee of Huaiâ€™an First Peopleâ€™s Hospital, Nanjing Medical University (Huaiâ€™an, China) (NJ-2018HA-0512).

### Real-time quantitative reverse transcription polymerase chain reaction test

We used TRIzolâ„¢ (Roche Diagnostics, Mannheim, Germany) to extract total RNA (21). We used Transcriptor First Strand cDNA Synthesis kit (Roche Diagnostics, Mannheim, Germany) to generate cDNA with 2 Âµg of each RNA sample. The LightCycler 480 SYBRÂ® Green 1 Master Mix (Roche Diagnostics) was used to perform polymerase chain reaction (PCR), with *GAPDH* as the reference gene. The primers (Sangon Biotech, Shanghai, China) used are listed below:

*TNFÎ±*:

F: CATCTTCTCAAAATTCGAGTGACAA

R: TGGGAGTAGACAAGGTACAACCC

*IL-1*:

F: CCGTGGACCTTCCAGGATGA

R: GGGAACGTCACACACCAGCA

*IL-6*:

F: AGTTGCCTTCTTGGGACTGA

R: TCCACGATTTCCCAGAGAAC

*GAPDH*:

F: ACTCCACTCACGGCAAATTC

R: TCTCCATGGTGGTGAAGACA

### Enzyme-linked immunosorbent assay

Tumour necrosis factor alpha (TNFÎ±), interleukin (IL)- 1, and IL-6 were detected with ELISA kits (BioLegend, San Diego, CA, USA) (22) and an ELISA plate reader (Synergy HT, BioTek, VT, USA) at an optical density of 450 nm.

### Oxidative stress

The amount of oxidative stress generated was measured as reported by Gu (23). We used 2Ê¹,7Ê¹-dichlorofluorescein diacetate (DCFH-DA) to detect reactive oxygen species (ROS) levels in the cells with an ELISA plate reader. Total superoxide dismutase (SOD), glutathione peroxidase (GDPs) and NADPH oxidase were with commercial kits (Beyotime, Beijing, China). 

### Nitric oxide production

We used a Griess reaction assay (Cayman Chemical, Ann Arbor, MI, USA) to detect NO production in the ECs in each group (8).

### Terminal deoxynucleotidyl transferase dUTP nick end labelling staining

ECs were fixed and permeabilized, then stained with the TUNEL reaction mixture to label the apoptotic cells. The nuclear was stained by 4â€™,6-Diamidino-2-Phenylindole, Dihydrochloride (DAPI) (21). 

### Western blot

We used SDS-PAGE to isolate the proteins (21). The following primary antibodies were used at a 1:1000 dilution: anti-NGAL, anti-Bax, anti-Bcl-2, anti-cytochrome C, anti-iNOS, anti-nNOS, anti-eNOS, anti-KEAP1, anti-NRF2 and anti-GAPDH (all purchased from Cell Signaling Technology, Danvers, MA, USA). Enhanced chemiluminescence (ECL) reagents (Bio-Rad, Hercules, CA, USA) with a ChemiDoc MP Imaging System (Bio-Rad) were used to scan the blots. The reference protein was GAPDH.

### Immunofluorescence

Cells were analysed for NRF2 nuclear translocation by immunofluorescence. The cells were fixed with 4% polyformaldehyde, permeabilized in 0.1% Tritonâ„¢ X-100 and incubated with anti-NRF2 antibody (Abcam, USA). The cells were then incubated with an Alexa FluorH 568 goat IgG (Invitrogen, Carlsbad, CA, USA) secondary antibody. The nucleus was stained with DAPI (Invitrogen, USA).

### Statistical analysis

**Fig.1:**
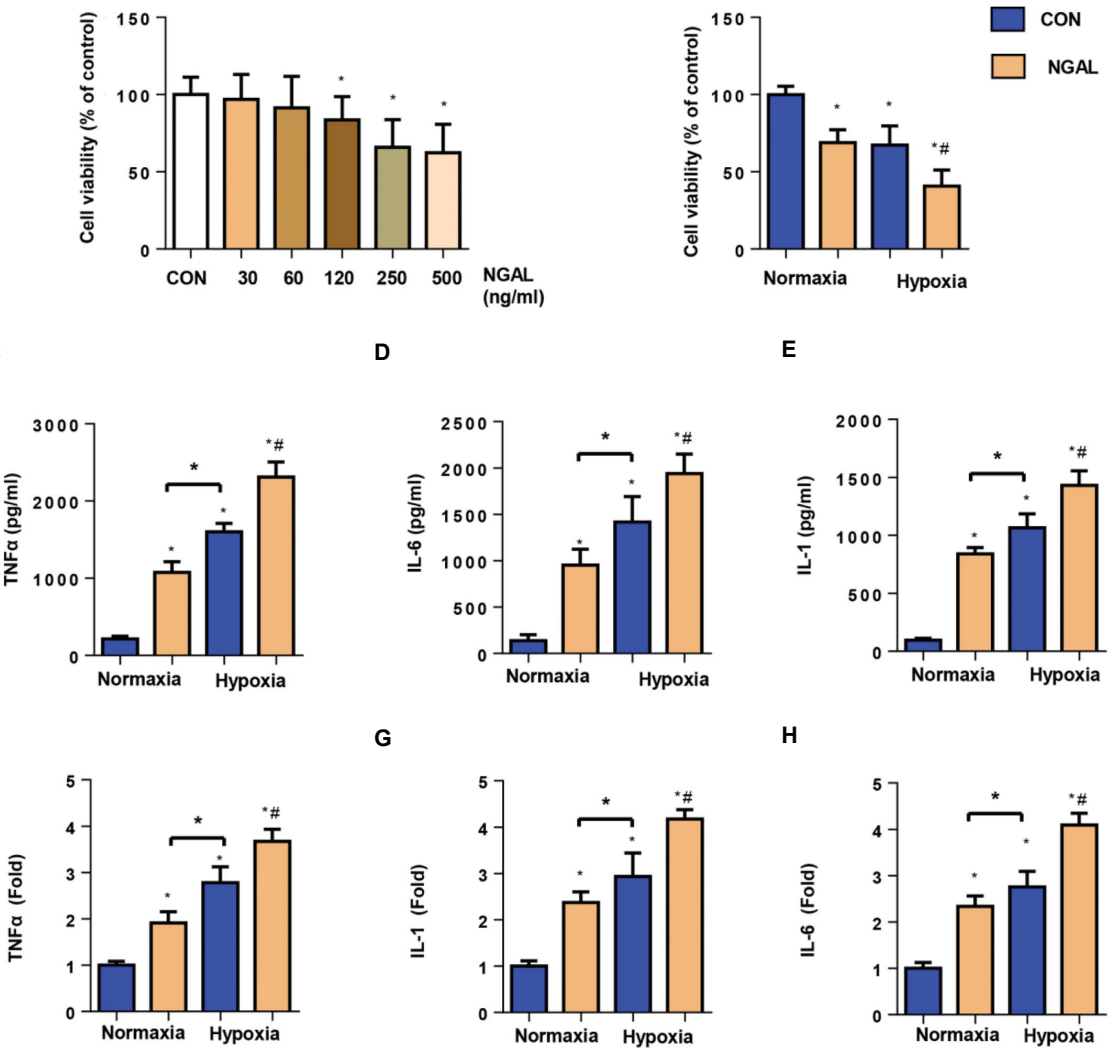
NGAL increases EC inflammation under hypoxia. **A., B. **Cell viability detected by the MTT assay under normoxic or hypoxic conditions for 24 hours with or without NGAL treatment (A: 30, 60, 120, 250, 500 ng/ml; B: 500 ng/ml, n=6). **C-E.** Release of pro-inflammatory cytokines in ECs under normoxic or hypoxic conditions for 24 hours with or without NGAL treatment (500 ng/ml, n=6).** F-H.** mRNA expression levels of pro-inflammatory cytokines in ECs (n=6). NGAL; Neutrophil gelatinase-associated lipocalin, EC; Endothelial cell, MTT; 3-(4,5-dimethylthiazol-2-yl)-2,5-diphenyltetrazolium bromide, Con; Control, TNFÎ±; Tumour necrosis factor alpha, IL; Interleukin, *; P<0.05 vs. normoxia-con, and ^#^ ; P<0.05 vs. hypoxia-con.

SPSS version 19.0 (SPSS Inc., Chicago, IL, USA) was used for statistical analysis. Data were expressed as mean Â± standard error. The unpaired studentâ€™s t test was performed to determine differences between two groups. One-way ANOVA followed by Tukeyâ€™s post-hoc test was used to analyse differences among groups, including the data in Figures 1A. Two-way ANOVA followed by Tukeyâ€™s post-hoc test was used to analyse the differences among the groups, including data in Figures 1B-H. P<0.05 indicated statistical significance.

## Results

### Neutrophil gelatinase-associated lipocalin increases endothelial cell inflammation under hypoxia

We treated ECs with different concentrations of NGAL (30, 60, 120, 250 and 500 ng/ml) and assessed them with the MTT assay to detect the influence of NGAL on cell viability. As shown in Figure 1A, cell viability was not significant between the 30 and 60 ng/ml NGAL and control groups, whereas cell viability decreased gradually in the 120, 250 and 500 ng/ml NGAL treatment groups compared with the control group. The cells were also exposed to hypoxia for 24 hours to elucidate the functional role of NGAL in hypoxia-induced injury in ECs. As shown in Figure 1B, cell viability decreased in the hypoxia group, and NGAL (500 ng/ml) treatment exaggerated these changes. Cell inflammation was detected by transcription and secretion of pro-inflammatory factors TNFÎ±, IL-1 and IL-6. As shown in Figure 1CH, NGAL increased mRNA expression and secretion of pro-inflammatory factors under normal as well as hypoxic conditions when compared with those in the corresponding control group.

### Neutrophil gelatinase-associated lipocalin accelerates endothelial cell imbalance of the redox system

**Fig.2:**
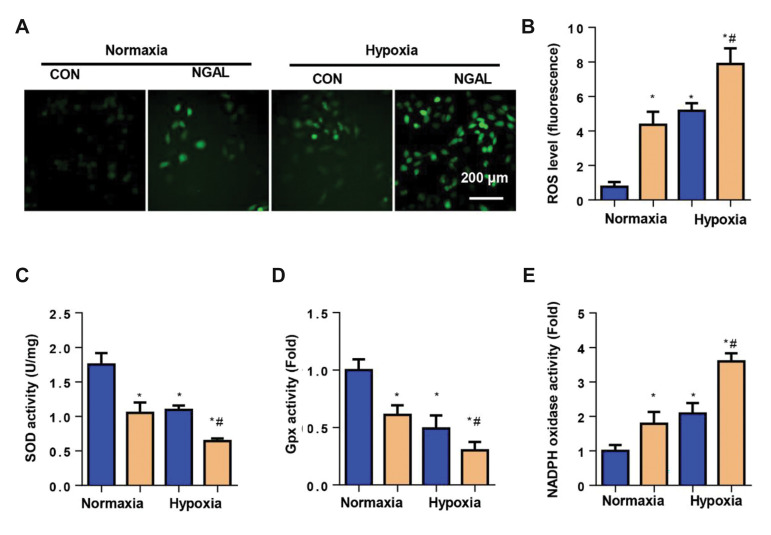
NGAL accelerates imbalance of the redox system in ECs. **A., B.** ROS levels in ECs under normoxia or hypoxia for 24 hours with or without NGAL treatment (500 ng/ml), detected by DCFH-DA (n=6). **C. **SOD activity. **D.** Gpx activity. **E.** NADPH oxidase activity in ECs (n=6). NGAL; Neutrophil gelatinase-associated lipocalin, EC; Endothelial cell, Con; Control, ROS; Reactive oxygen species, DCFH-DA; 2Ê¹,7Ê¹-dichlorofluorescein diacetate, SOD; Superoxide dismutase, and Gpx; Glutathione peroxidase, *; P<0.05 vs. normoxia-con, and ^#^ ; P<0.05 vs. hypoxia-con.

Redox imbalance is a major cause of injury from cell inflammation during hypoxia. Therefore, we determined the redox status of ECs in both normoxic and hypoxic cells treated with NGAL. As observed by DCFH fluorescence, the ROS level increased sharply in the NGAL treatment group compared to the control group of normoxic cells. NGAL also accelerated hypoxia-induced ROS production (Fig .2A, B). The activities of antioxidases SOD and Gpx, along with NADPH oxidase were determined. We observed reduced activities of SOD and Gpx and increased activity of NADPH oxidase in NGAL-treated ECs under both normoxic and hypoxic conditions compared with the corresponding control group (Fig .2C-E).

### Neutrophil gelatinase-associated lipocalin increases endothelial cell apoptosis under both normoxic and hypoxic conditions

**Fig.3:**
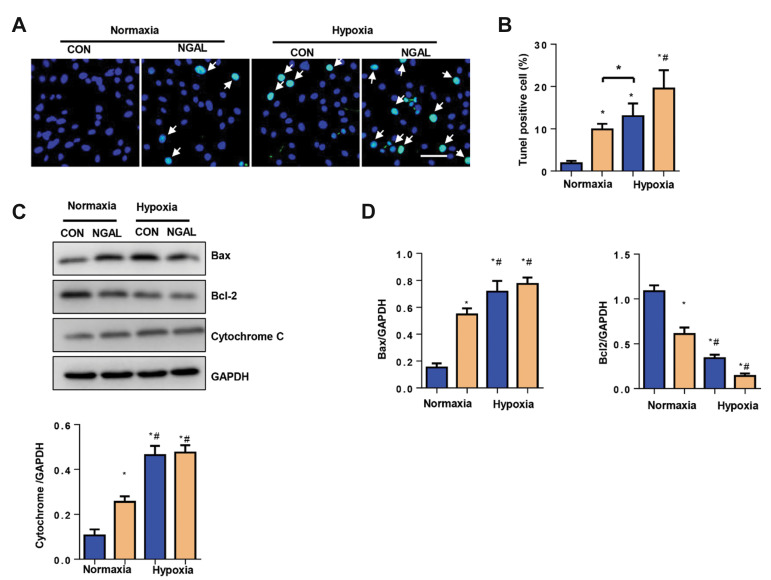
NGAL increases EC apoptosis under both normoxic and hypoxic conditions. **A., B.** Apoptosis rates in ECs under normoxia or hypoxia for 24 hours with or without NGAL treatment (500 ng/ml), detected by the terminal deoxynucleotidyl transferase dUTP nick end labelling (TUNEL) assay (n=6) (scale bar: 100 Î¼m). **C., D. **Protein levels of Bax, Bcl-2 and cytochrome C in ECs (n=6). White arrow; Tunel positive cells, NGAL; Neutrophil gelatinase-associated lipocalin, EC; Endothelial cell, Con; Control, TUNEL; Terminal deoxynucleotidyl transferase dUTP nick end labelling, *; P<0.05 vs. normoxia-con, and ^#^ ; P<0.05 vs. hypoxia-con.

Increased cell inflammation and oxidative stress cause cell apoptosis. Hence, we used the TUNEL assay to detect cell apoptosis. There was an increase in apoptosis rate in NGAL-treated ECs, even under normoxic conditions. Hypoxia increased the apoptosis rate; NGAL remarkably exaggerated this increased apoptosis rate under the hypoxic condition (Fig .3A, B). Apoptosis markers were also detected by Western blot analysis. As shown in Figure 3C and D, increased Bax and cytochrome C were observed in two NGAL treatment groups compared with those in the control and hypoxia groups. Bcl-2 decreased in the two NGAL treatment groups under normoxic and hypoxic conditions.

### Neutrophil gelatinase-associated lipocalin affects the endothelial nitric oxide synthase-nitric oxide-nuclear factor erythroid-2-related factor 2 axis

**Fig.4:**
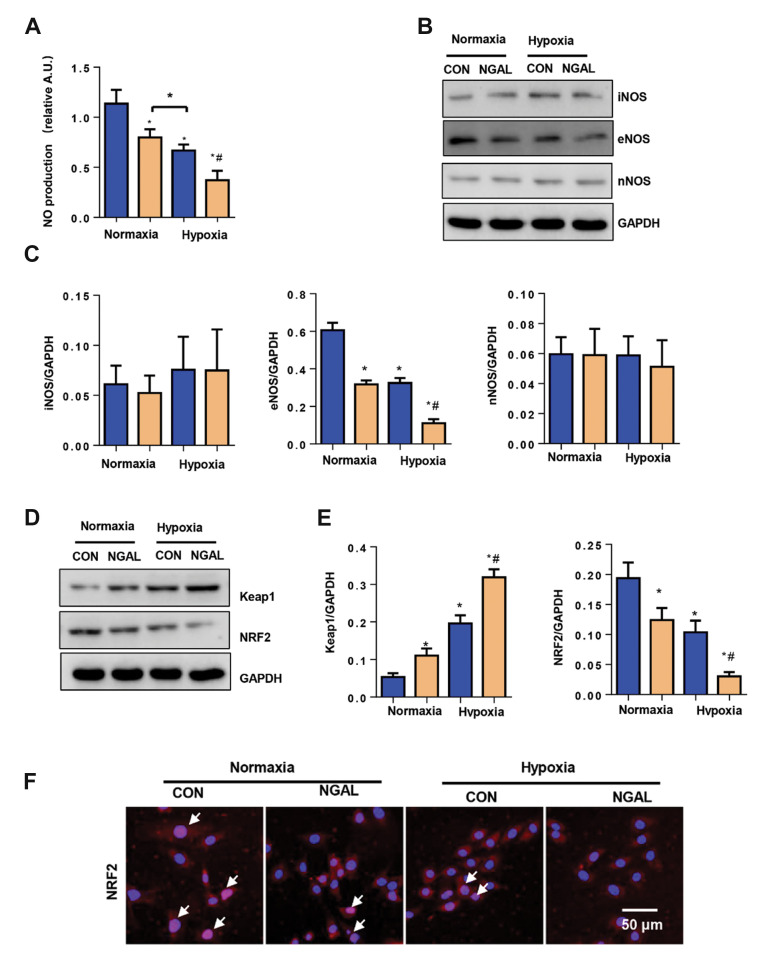
NGAL affects the eNOS-NO-NRF2 axis. **A.** NO level in ECs under normoxia or hypoxia for 24 hours with or without NGAL treatment (500 ng/ml, n=6).** B., C.** Protein levels of iNOS, nNOS and eNOS in ECs (n=6). **D., E. **Protein levels of KEAP and NRF2 in ECs (n=6). **F. **NRF2 staining in ECs (n=6). White arrow: NRF2 nuclear transition, NGAL; Neutrophil gelatinase-associated lipocalin, EC; Endothelial cell, Con; Control, eNOS-NO-NRF2; Endothelial nitric oxide synthase-nitric oxide nuclear factor erythroid-2-related factor 2, iNOS; Inducible NOS, nNOS; Neuronal NOS, *; P<0.05 vs. normoxia-con, and # ; P<0.05 vs. hypoxia-con.

We then screened the underlying mechanism that mediates the functional role of NGAL in an EC injury. We first screened the secretion of molecules by ECs and found that NO sharply reduced in the NGAL treatment group under both normoxia and hypoxia (Fig .4A). Then, we detected expression levels of the three NOS. Unexpectedly, iNOS and nNOS were not significantly altered between the NGAL treatment and control groups under both normoxic and hypoxic conditions, whereas eNOS was reduced in the two NGAL groups compared with the control and hypoxia groups (Fig .4B, C). NO is a small gas molecule that can function in all cell types of the heart; therefore, we sought to determine if this eNOS-NO pathway function directly affects the redox system. Thus, as a typical redox molecule, NRF2 was detected. Interestingly, we found that the KEAP1, a repressor of NRF2 was increased in the NGAL treatment group and NRF2 was reduced in the NGAL treatment group under both normoxic and hypoxic conditions (Fig.4D, E). Immunofluorescence staining to detect nuclear translocation of NRF2 results showed a decrease in NRF2 nuclear translocation in the two NGAL treatment groups (Fig .4F).

### Nuclear factor erythroid-2-related factor 2 overexpression blocks the functional role of neutrophil gelatinase-associated lipocalin

**Fig.5:**
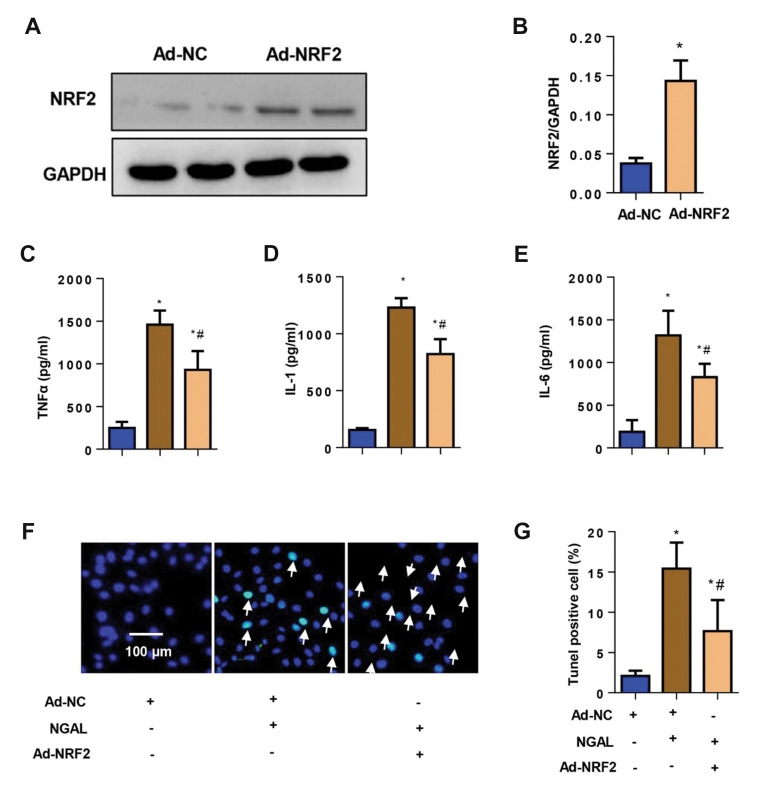
NRF2 overexpression blocks the functional role of NGAL. Cells were treated with NGAL (500 ng/ml) for 24 hours and then transfected with Ad-NRF2 for 8 hours. **A., B. **Protein level of NRF2 in ECs transfected with Ad-NRF2. **C-E. **Release of pro-inflammatory cytokines in ECs (n=6).** F.** and **G. **Apoptosis rates in ECs (n=6). White arrow; Tunel positive cell, NRF2; Nuclear factor erythroid-2-related factor 2, Ad; Adenovirus, NC; Negative control, NGAL; Neutrophil gelatinase-associated lipocalin, EC; Endothelial cell, TNFÎ±; Tumour necrosis factor alpha, IL; Interleukin, *; P<0.05 vs. Ad-NC, and ^#^ ; P<0.05 vs. NGAL.

**Fig.6:**
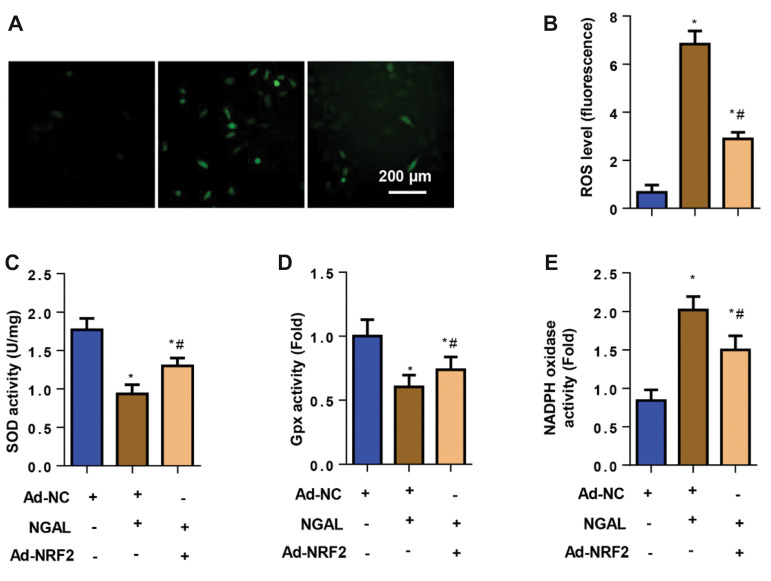
Cells were treated with NGAL (500 ng/ml) for 24 hours and then transfected with Ad-NRF2 for 8 hours. **A., B.** ROS levels in ECs (n=6). **C.** SOD activity. **D.** Gpx activity. **E. **NADPH oxidase activity in ECs (n=6). NGAL; Neutrophil gelatinase-associated lipocalin, EC; Endothelial cell, Ad-NRF2; Adenovirus-nuclear factor erythroid-2-related factor 2, ROS; Reactive oxygen species, SOD; Superoxide dismutase, Gpx; Glutathione peroxidase, NC; Negative control, *; P<0.05 vs. Ad-NC, and ^#^ ; P<0.05 vs. NGAL.

We next confirmed whether NRF2 overexpression could block the NGAL functional role in ECs. Ad-NRF2 was used to overexpress NRF2. Figure 5A and B show a sharp increase in NRF2 protein level by Ad-NRF2 transfection. ECs were treated with NGAL for 24 hours and then transfected with Ad-NRF2 for 8 hours. As a result, NGAL-induced increased secretion of pro-inflammatory factors was counteracted by NRF2 overexpression (Fig .5C-E). EC apoptosis induced by NGAL treatment also decreased in the NRF2 overexpression group (Fig .5F, G). ROS level and NADPH oxidase activity decreased, whereas SOD and Gpx levels increased in the NRF2 overexpression group compared with those in the NGAL treatment group (Fig .6). These data indicate that NGAL causes EC injury by inhibiting the eNOS-NO-NRF2 axis.

## Discussion

NGAL plays a role in cardiac hypertrophy because it is a biomarker for many CVD (19), MI (24) and fibrosis (17). However, the effect of NGAL on ECs has not been elucidated. ECs are a major cell type in the heart tissue that participate in the formation of capillaries as well as in the secretion of many molecules that influence cardiomyocytes and fibroblasts (5, 6). In this study, we first explored the functional role of NGAL in ECs under hypoxia. Unexpectedly, we found that NGAL caused EC injury (inflammation, redox imbalance and apoptosis) even under normal oxygen supply conditions. NGAL exaggerated the hypoxia-induced EC injury. Second, we found that NGAL-induced EC injury relied on eNOS-NO-NRF2-mediated redox regulation in ECs.

NO is a small molecule polypeptide that maintains vasodilation. Clinically, nitroglycerin can be used to treat a variety of CVD by releasing NO (25). In addition, continuous inhalation of NO can improve symptoms in patients with persistent pulmonary hypertension (26). NO gas, nitrite, nitrate or NO donors have been shown to ameliorate reperfusion injuries in both animal and human experiments (27). Previous studies reported that NGAL could act as a biomarker to predict malignant events in various CVD, such as unstable plaques in patients with asymptomatic carotid stenosis (16), HF and cardiovascular events (14) in patients with stable coronary artery disease (15). Eilenberg et al. (28) found enhanced NGAL levels in ECs in human carotid atherosclerotic tissue. Human umbilical vein ECs treated with NGAL led to a great pro-inflammatory response. In our study, we found that NGAL caused MHECs injury by reducing NO production under normal as well as hypoxic conditions. This might be the core factor that mediates EC injury. long-term hypoxia can inhibit eNOS expression (29). Prolonged ischemia in the heart leads to reduced eNOS mRNA and protein levels (30). It has been reported that NGAL suppressed autophagy, which led to cardiomyocyte hypoxia injury (24). MartÃ­nez-MartÃ­nez et al. (17) reported that NGAL-induced cardiac remodelling was relied on NF-kappa B pathway. However, in our study, we observed increased eNOS expression levels after 24 hours of hypoxia in the ECs. These data were consistent with those of a previous study (29). Litte reported the effect of nNOS on ischemia (30). In our study, we did not report any changes in the expression level of nNOS in ECs. iNOS increases in the presence of acute injury and inflammation (31). We found an increase in iNOS, but it was not statistically different. A previous study has reported that NO production is elevated in ischemic heart tissue (32). However, we found that NO production by ECs decreased after 24 hours of hypoxia. This inconsistency may be due to the release of NOS-NO in other cell types of the heart tissue, such as cardiomyocytes and fibroblasts. We observed that NGAL merely decreased the expression of eNOS, but not iNOS and nNOS, which caused a further reduction in NO release.

NRF2 is an important transcription factor that reduces ROS and promotes resistance of the body to harmful external stimuli. Under physiological conditions, KEAP1 in the cytoplasm is linked to NRF2 inactivation (33). The results of a study have shown that upregulation of eNOS results in increased nitrosylation of KEAP1 (34), which leads to the degradation of NRF2 and subsequent release of NRF2 into the nucleus. In the nucleus it recognizes and binds to a series of AU-rich elements (AREs) to activate the expression of certain corresponding phase-II detoxification enzyme genes and induces the expressions of SOD, catalase (CAT) and glutathione (GSH), leading to a balanced redox system (35). In our study, we found that hypoxia decreased the expression and nuclear translocation of NRF2. NGAL increased the expression of KEAP1 and reduced NRF2 nuclear translocation, which caused an imbalance in the redox system and increased ROS level in ECs. We found that 500 ng/ml NGAL could induce ECs injury when the cells were exposed to normal oxygen levels. These deteriorating effects were mediated by a decrease in the level of NRF2 under physiological conditions. Moreover, NGAL could accelerate the deteriorating effects of hypoxia by further reducing NRF2 levels. NGAL treatment alone has been reported to suppress cardiomyocyte autophagy and induce cell apoptosis (24). Moreover, NGAL increased the numbers of injured cardiomyocytes under hypoxic conditions. This result was consistent with our finding that NGAL alone could induce and exaggerate the hypoxia injury to these ECs. Our data suggest that NGAL is a strong pro-EC injury factor; thus, decreasing the level of NGAL may be a treatment target for CVD.

## Conclusion

We first found that NGAL exaggerated the EC injuries under both normoxic and hypoxic conditions. The augmenting role of NGAL in the EC injury was primarily dependent on the eNOS-NRF2 signalling pathway.
